# Cell Structure in LPBF 316L—Microstructural Heterogeneity, Thermal Stability, and Mechanical Properties

**DOI:** 10.3390/ma18030475

**Published:** 2025-01-21

**Authors:** Jayant Barode, Marco Brander, Tianbo Yu, Venkata Karthik Nadimpalli, Dorte Juul Jensen, Xiaobo Wang

**Affiliations:** Department of Civil and Mechanical Engineering, Technical University of Denmark, 2800 Kongens Lyngby, Denmark; mabran@dtu.dk (M.B.); tiyu@dtu.dk (T.Y.); vkna@dtu.dk (V.K.N.)

**Keywords:** 316L stainless steel, laser powder bed fusion, cell structure, microstructural heterogeneity, thermal stability, electron microscopy, dislocation density

## Abstract

The microstructure of additively manufactured 316L stainless steel is hierarchical, and on a fine scale, it contains cell structures and dislocations. These microstructures define the mechanical properties, and it is thus of importance to quantify them and understand their thermal stability. This study investigates the heterogeneity of the microstructure in laser powder bed-fused 316L with a focus on variations in the cell and dislocation structures through the sample thickness along the build direction. While at the coarse scale the microstructure is rather homogeneous throughout its thickness, there are significant variations in the dislocation network, highlighting a higher dislocation density near the bottom layers than near the top. Furthermore, post-processing heat treatment at 500 °C and 800 °C reveals different stabilities of the cell structures, with significant cell dissolution at 800 °C, particularly at the top of the build. Microhardness measurements corroborate these findings, showing higher hardness in the bottom layers across all conditions, e.g., an increase in hardness from 225 HV to 236 HV is observed in the as-built condition. These results underpin the suggestion that significant microstructural heterogeneity may exist through the thickness in as-built parts, which affects the mechanical properties and subsequent heat treatments.

## 1. Introduction

Microstructure is crucial in defining the properties of metallic materials. The microstructure depends on the manufacturing and post-processing processes. Laser powder bed fusion (LPBF) is a popular additive manufacturing technique, which creates 3D components in a layer-by-layer fashion through the localized melting of powders, using a computer-controlled laser beam [1]. Various alloys are suitable for LPBF. Among them, austenitic 316L stainless steel (SS) is frequently used. This alloy is well-known from conventional manufacturing and is applied widely for food and kitchen appliances, medical tools, nuclear power plants, oil rigs, etc. [2]. LPBF leads to microstructures that are quite different from those known from conventional manufacturing [2,3], and the temperature gradient (*G*) and crystal growth rate (*R*) play significant roles in determining the morphology of the rapid solidification microstructures in LPBF [1].

**Figure 1:**
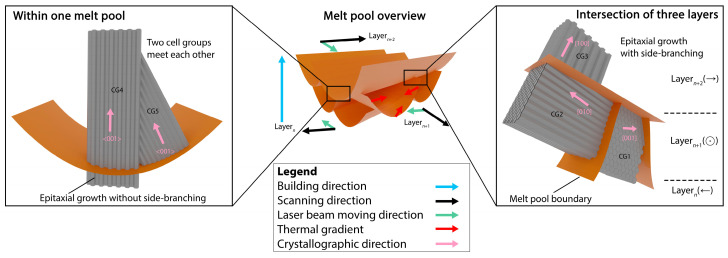
Schematic illustration of the LPBF 316L SS microstructure, with a focus on melt pools and cell groups (CGs) adapted from ref. [5]. The sketch shows melt pools within three printed layers. The building direction, scanning direction, laser beam moving direction, thermal gradients, and crystallographic direction are indicated by colored arrows. The sketches in the two black boxes are zoom-in images of regions near a melt pool boundary intersection and the bottom of the melt pool, respectively, showing the epitaxial growth of cells with or without side-branching. The scanning direction for each layer is shown in brackets in the black box to the right.

The LPBF of 316L SS usually produces a hierarchical microstructure spanning multiple length scales. At the macro-scale, due to localized melting and solidification, the LPBF process produces melt pools, whose geometry highly depends on the printing parameters. Within the melt pool, there are grains that typically exhibit an elongated morphology based on the local thermal gradient [2,3,4]. An individual grain contains several cell groups (CG), each consisting of a bunch of cells with nearly the same crystallographic orientation. The cells are believed to have an elongated n-gonal, prism-like 3D morphology. In 2D, they thus appear as equiaxed, extended, or columnar, based on their relationship with the observation plane [5]. The cells grow epitaxially and are in 316 SS aligned along one of the <001> crystallographic directions, depending on the thermal gradient. Due to the complexity of local heat flow during the LPBF process, cells are also observed to side-branch [6]. This usually happens at the fusion line when the new melt pool is formed. A shift in the local thermal gradient away from its previous growth direction may cause a change in the growth direction of the cell axis by 90°, aligning to an alternative <001> direction, which is the closest to the thermal gradient [5]. A schematic is shown in Figure 1 demonstrating the hierarchical microstructural features in LPBF 316L SS. For each cell, most of the dislocations are entangled in the cell wall, while the rest are distributed in the cell interior, forming loose dislocation boundaries. Together, these dislocations form a bamboo-like dislocation network [5].

While much focus has been on the coarser scale features of the microstructure, far less attention has been devoted to the cell’s structure and dislocations. It is reported that the cell structure in 316L SS may be associated with elemental segregation as well as high dislocation densities at the cell walls [3]. These, to some extent, overlap, and, based on the individual dominance, they are in the literature termed either as segregation networks or dislocation networks. There have primarily been two hypotheses for the formation of the high-density dislocation networks along the cell walls. One hypothesis is that they form during the final stages of rapid solidification and do not significantly evolve under the repeated thermal cycles of LPBF [7]. The other hypothesis is that their formations relate to this repeated thermal cycling [2]. Recently, a novel hypothesis suggested that they primarily originate from rapid solidification and subsequently evolve during repeated thermal cycles [5].

The microstructures are responsible for the superior strength and ductility of the LPBF-316L SS [2]. Multiple strengthening mechanisms contribute. For example, the cell structure, with its high dislocation density at the cell walls, acts as an effective trapping site and an obstacle for the dislocation motion [8], and the yield strength has been shown to be inversely proportional to the cell size [3,9]. Solutes, segregated along the cell walls, have been reported to produce coherency strains due to the chemical misfit, which can further resist dislocation motion [10]. Oxide inclusions, also present along the cell walls, are furthermore reported to increase the strength by Orowan strengthening [2,11]. In addition to the strength, cell structures in LPBF 316L SS have been reported to contribute to better ductility [2,12] and, in some cases, higher corrosion resistance [9] than in the conventionally wrought counterparts.

As discussed in refs. [13,14], it is thus of interest to study the printed microstructures in much more detail. In particular, we find it important to study the fine-scaled cell structures and also to focus on the dislocations inside the cells. Although the dislocation density in the interior is far less than that in the cell walls, they may still contribute to the mechanical properties. Furthermore, it is of interest to understand the thermal stability of these cell structures, as printed parts may be in use at elevated temperatures. Considering the latter, quite different results are reported in the literature for annealing at temperatures in the range of 600–1200 °C [4,15,16,17]; for example, Funch et al. did not observe the cells after annealing at 900 °C for 1 h [4], while Roirand et al. reported cells even after annealing at 900 °C for 2 h [16,17].

With the aim of clarifying this and understanding if a possible heterogeneity through the printed sample thickness may explain different observations of the cell structure’s thermal stability, in this work, we investigate the microstructural heterogeneity of the cell structure and the interior dislocations along the building direction of as-built 316L SS parts and follow their evolution during post-processing heat treatments. Finally, we relate the observations to hardness measurements to evaluate their effects on the mechanical properties.

## 2. Materials and Methods

### 2.1. Printing and Post-Processing Heat Treatment

**Figure 2:**
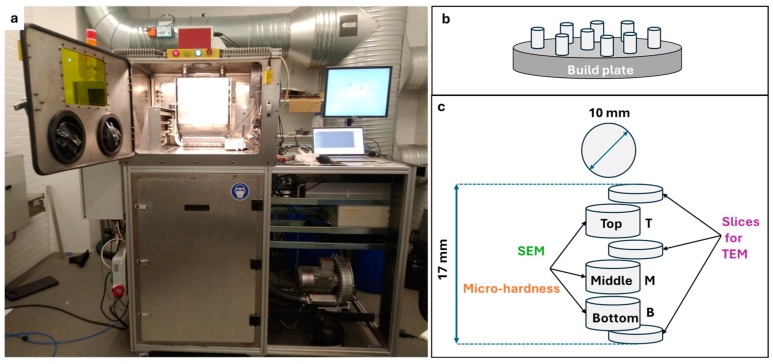
(**a**) Open-architecture LPBF machine used in this work, (**b**) schematic of the printed cylindrical samples, and (**c**) slices extracted along the build direction of the cylindrical samples.

Gas-atomized 316L SS powder was purchased from Höganäs AB, Höganäs, Sweden. The chemical composition of the powder is listed in Table 1. The D_10_, D_50_, and D_90_ of the powder particles are 25.1 µm, 41.3 µm and 64.8 µm, respectively. Cylindrical samples (diameter = 10 mm and height = 18 mm) were printed in an open architecture LPBF machine developed at the Technical University of Denmark (DTU), Lyngby, Denmark [18] (Figure 2a). Using this LPBF machine, our previous work focused on optimizing the processing parameters for 316L [19]. The printing parameters in the present work were selected based on the relative density, leading to almost fully dense parts of ~99% at a volumetric energy density of 76.9 J/mm^3^. The selected values of all the printing parameters are given in Table 2.

As the purpose of this work is to quantify the through-thickness heterogeneity of the cell structure along the build direction and study the thermal evolution, the experiment was designed to investigate the sample slices from the top (T), middle (M), and bottom (B) of the as-built sample, as well as of heat-treated samples (see Figure 2b,c). The thermal stability of the cell structure was investigated after annealing at 500 °C and 800 °C for 2 h in air, followed by furnace cooling. These two temperatures, a low and an intermediate one, were chosen in order to track the evolution of the cell structures. Higher temperatures (above 1000 °C) have been reported to result in the total dissolution of the cell structure [16] and are thus not of relevance for the present work.

The as-built samples were cut from the build plate using electric discharge machining (EDM), while a Struers Accutom rotary cutter was used to prepare the top, middle, and bottom sample slices.

### 2.2. Characterization

The thicker sample slices (see Figure 2c) were embedded in conductive resin for scanning electron microscopy (SEM) and microhardness characterization. Thinner slices, with thicknesses of 250 µm, were prepared for transmission electron microscopy (TEM) observations.

The embedded samples for SEM were polished in a Struers Rotopol-21 polishing machine (Struers A/S, Ballerup, Denmark). They were mechanically polished using a 1-μm diamond suspension, followed by OP-S polishing. To reveal the cell structure, the prepared samples were etched using a solution with HF: HNO_3_: H_2_O = 2:8:90. SEM imaging was performed in a Zeiss Sigma 300 microscope (Carl Zeiss Microscopy Deutschland GmbH, Oberkochen, Germany) equipped with Energy Dispersive Spectroscopy (EDS) at 20 kV. Microstructural analysis was performed using the ImageJ 1.53k software [20]. For TEM sample preparation, the thin sample slices were polished to thicknesses of around 100 µm, and then discs of 3 mm in diameter were punched out and further polished down to 90 µm in thickness. Finally, twin-jet electropolishing was performed by a mixture consisting of 90% ethanol and 10% perchloric acid, at –20 °C [21]. The TEM samples were characterized using a JEOL JEM-2100 microscope (JEOL Ltd., Tokyo, Japan) at 200 kV, and a double-tilt sample holder was used to enable a clear view of dislocations. The microhardness tests of the embedded samples were performed using a Struers DuraScan tester (Struers A/S, Ballerup, Denmark) following the standard ASTM E384-22 [22]. A load of 0.5 kg was used, and a minimum of 12 indentations were made on each sample. 

## 3. Results

### 3.1. Microstructure

**Figure 3:**
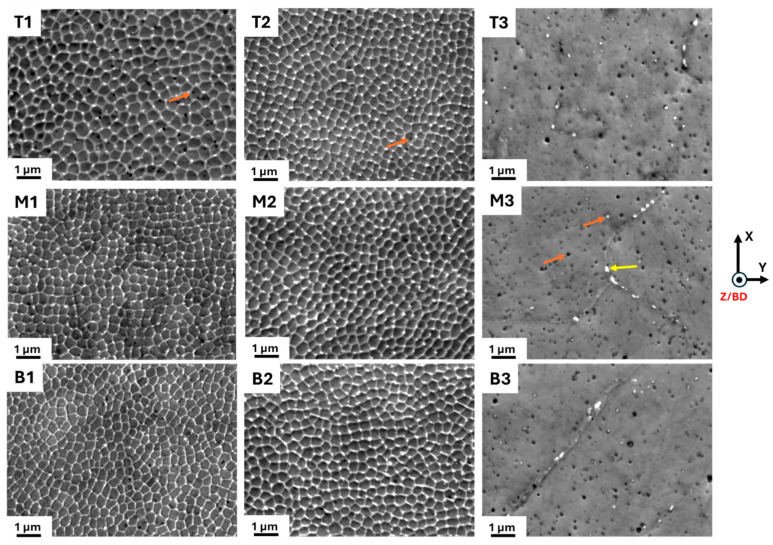
SEM micrographs of the top (**T**), middle (**M**), and bottom (**B**) parts of the samples, taken perpendicular to the build direction in the as-built (**T1**,**M1**,**B1**), 500 °C-2 h (**T2**,**M2**,**B2**), and 800 °C-2 h (**T3**,**M3**,**B3**) conditions. Yellow and orange arrows mark grain boundary precipitates (non-spherical white) and locations of oxide inclusions (spherical black and white spots), respectively.

As described above in the introduction, the cells have elongated, n-gonal 3D prism-like morphologies: see Figure 1. In the present work, we focused on analyzing the cells that are perpendicular to the prism length, i.e., in grains/sample positions where we see them as equiaxed cells. Figure 3 shows SEM micrographs of the as-built (Figure 3 (T1,M1,B1)), 500 °C-2 h (Figure 3(T2,M2,B2)), and 800 °C-2 h (Figure 3(T3,M3,B3)) samples observed at the top, middle, and bottom, respectively. In the as-built condition, a clear cell structure is observed in agreement with many previous observations. Upon heat treatment at 500 °C-2 h, a clear cell structure is still apparent. However, after 800 °C-2 h annealing, the cell structure disappears. An estimation of the mean cell size was obtained using the line intercept method by ASTM E112 [23]. Seven random lines were drawn on the micrograph. The number of interceptions was counted as 1 and 1.5 for the cell boundary and triple junctions, respectively. Three micrographs for each condition were utilized. The resulting cell sizes are reported in Table 3 and are in the range of 314 nm to 385 nm. It appears the cell size increases from the bottom to the top in the as-built condition, which may be a correct trend. However, given the standard deviation, it is fair to conclude that neither the build height nor annealing at 500 °C-2 h has a significant effect on the cell size. The variations reported in Table 3 merely reflect the local variations within a melt pool.

**Figure 4:**
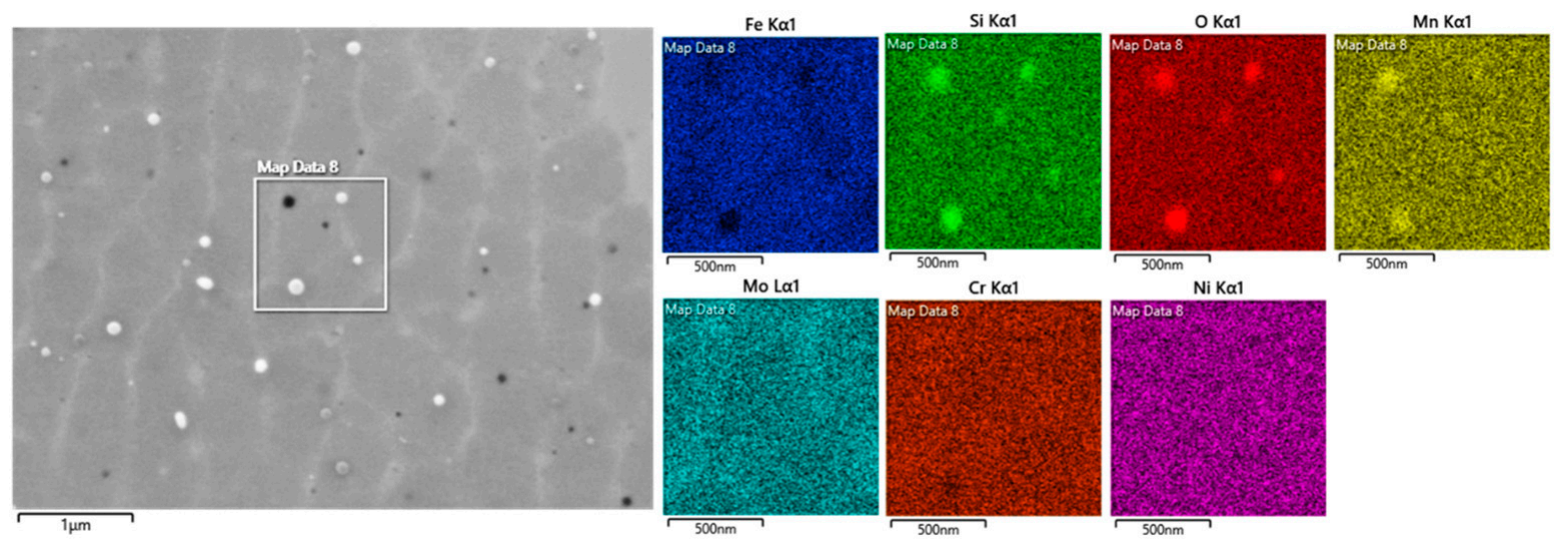
SEM–EDS map revealing the cell boundaries and spherical particles in the as-built-middle sample.

In the as-built and 500 °C-2 h states, fine spherical particles were observed as black and white spots along the cell walls and within the cells (Figure 3(T1,T2)). Note, however, that some of the black spots are small holes on the surface. It is difficult to distinguish whether they are caused by the dropout of particles during etching or whether they are voids that are generated during LPBF processing. An EDS map of a non-etched sample is shown in Figure 4. The spherical white and black particles were found to be enriched with Mn, Si, and O, confirming that these are Mn- and Si-rich oxides, as reported elsewhere [24]. The cell walls show a weak segregation of Mo and Cr.

After 800 °C-2 h annealing, the oxide particles substantially increased in number and coarsened from ~50 nm to ~100 nm. However, no significant difference in their average sizes along the build height was found (Table 3). In addition to the spherical Mn- and Si-rich oxide particles (orange arrows), there are also non-spherical white particles (Figure 3(T3,M3,B3)). They are located predominantly along the grain boundaries in the 800 °C-2 h sample. These white particles are reported to be Cr- and Mo-rich intermetallic, identified as σ phase [24,25].

**Figure 5:**
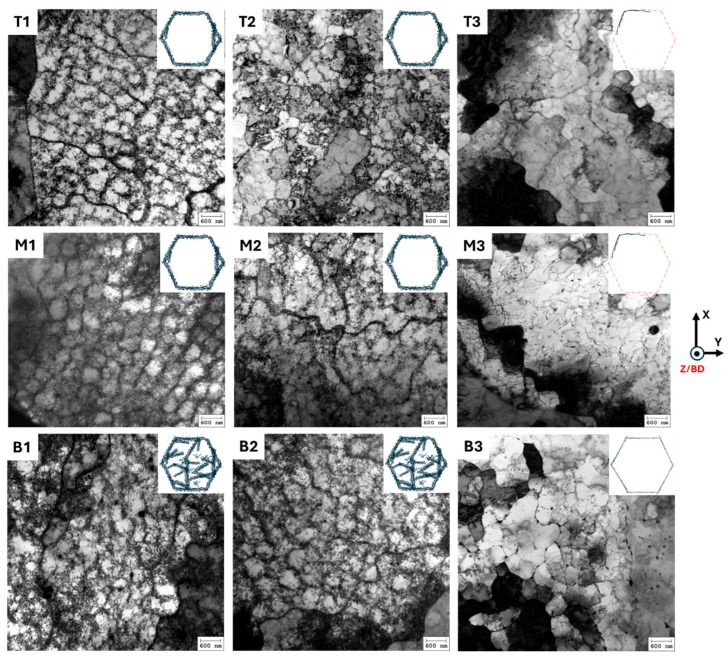
TEM micrographs taken perpendicular to the build direction in the as-built (**T1**,**M1**,**B1**), 500 °C-2 h (**T2**,**M2**,**B2**), and 800 °C-2 h (**T3**,**M3**,**B3**) conditions (inset shows schematics of dislocation network of a cell).

With SEM, it is typically not possible to observe individual dislocations. Thus, in order to study the dislocations at the cell walls and in the cell interior in more detail, TEM was employed. Figure 5 displays the TEM micrographs for the as-built, 500 °C-2 h, and 800 °C-2 h conditions at various heights. These observations reveal significant heterogeneities along the build height for all the conditions. While the top and middle samples have similar structures, they are distinctly different from the bottom ones. In the as-built condition, the bottom sample (Figure 5(B1)) was observed to have a higher dislocation density than those in the top and middle samples (Figure 5(T1)). At the bottom, a rather high dislocation density is present along the cell walls as well as within the cell interior, whereas at the top and middle, the dislocations are primarily present along the cell walls, with only a few dislocations in the cell interior. Similar observations were found in the 500 °C-2 h condition: see Figure 5 (T2,M2,B2).

After annealing at 800 °C-2 h (see Figure 5(T3,M3,B3)), a much thinner dislocation network was observed, indicating the annihilation of the dislocations. Even though the dislocation density was reduced considerably, a weak cell structure is still visible (too weak to be seen by SEM). Similar to the as-built and the lower-temperature annealed (500 °C-2 h) samples, a higher dislocation density was found near the bottom of the build. At the top and middle (Figure 5(T3,M3)), the cells have less well-defined cell walls, whereas at the bottom (Figure 5(B3)), the cell structure is clearly visible.

### 3.2. Microhardness

**Figure 6:**
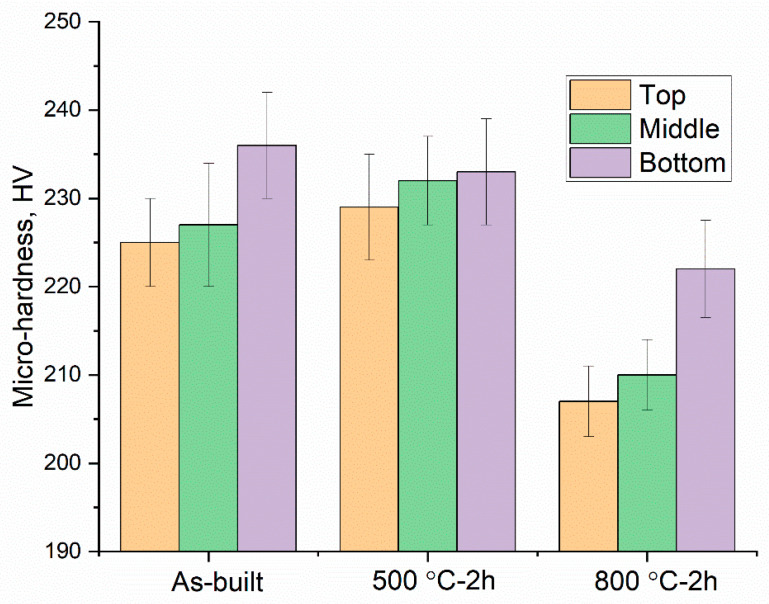
Miro-hardness values at various locations along the build height in the as-built, 500 °C-2 h, and 800 °C-2 h conditions.

The microhardness values for the as-built, 500 °C-2 h, and 800 °C-2 h conditions at the three sections are shown in Figure 6. All the conditions show a gradient in hardness along the build height, i.e., the bottom was found to be harder than the top. The hardness values of the as-built and 500 °C annealed samples are comparable (225–236 HV). After 800 °C-2 h annealing, the hardness was significantly reduced (207–222 HV). It is interesting to note that the hardness values of LPBF 316L in all the conditions are still higher than that of the wrought 316L (~200 HV) [26].

## 4. Discussion

### 4.1. Cell Structure Heterogeneity

The results show that there is a cell-structure heterogeneity along the build height in all the conditions, i.e., the as-built, 500 °C-2 h, and 800 °C-2 h (Figure 3 and Figure 5). In particular, the bottom of the build has a cell structure different from those of the top and middle, with a significantly higher dislocation density both in the cell walls and within the cell interior. The microhardness values highlight the importance of this, revealing higher hardness near the bottom of the build (Figure 6). The higher dislocation density at the bottom of the build may be due to the generation of high thermal stresses there [27] because of a steep thermal gradient that occurs when the first layers are printed on the relatively cooler build plate. Additionally, thermal mismatch between the build plate and deposited material could contribute, but this does not have a large effect on the present samples, as the build plate is also of 316L SS. Interestingly, the top and middle samples have similar cell structures (Figure 5). This suggests that the repeated thermal cycles might have an influence on the evolution of the dislocation network in the already solidified layers. However, only for a certain number of subsequent layers. After that, a given previously deposited layer might not reach a temperature high enough to influence the cell structure; for example, it was observed that at temperatures up to 500 °C, the cell structure remained stable.

The heterogeneity in the cell structure remains even after heat treatments of 500 °C-2 h and 800 °C-2 h. After 500 °C-2 h annealing, no noticeable change in the cell structure was observed, as compared to the as-built sample (Figure 3 and Figure 5(T2,M2,B2)). Similar observations were also reported elsewhere [3]. The usual temperature range for the cell dissolution has been reported to be 600–1200 °C [2,9]. Saeidi et al. [28] suggested that, in spite the high dislocation density in the cell walls in the LPBF samples, these cell structures exhibit good thermal stability due to their low energy configuration. Heavy elements such as Cr and Mo (Figure 4) that may be trapped at the cell walls could also improve their thermal stability at low-temperature annealing treatments [15,29].

In the present work, most of the cell structures disappeared either partially or completely after 800 °C-2 h annealing (Figure 5(T3,M3,B3)). The bottom sample retained well-defined cell walls (with a reduced thickness), as compared to the top and middle. Furthermore, the SEM micrographs indicate that the diffusion of segregated elements out of the cell walls may contribute to the annihilation of dislocations. The significant drop in the microhardness values (Figure 6) relative to the as-built and 500 °C-2 h samples is likely a synergetic effect of the loss of segregated elements and reduced dislocation density.

### 4.2. Oxide Inclusions and Precipitation

In the as-built and 500 °C-2 h annealed samples, Mn- and Si-enriched oxide inclusions were found at the cell walls and within the cells (Figure 3(T1,T2) and Figure 4). Multiple sources of oxygen can contribute to their formations in the as-built sample: (i) oxide inclusions in the powder particles pre-formed during inert gas atomization, (ii) the presence of oxygen in the chamber atmosphere during LPBF process, and (iii) moisture pickup from a humid atmosphere during powder storage [30].

After 800 °C-2 h annealing, numerous coarsened oxide particles formed in the cell interior: see Figure 3. To the best of our knowledge, no mechanism has been suggested for the evolution of oxide inclusions during the annealing of LPBF 316 L SS. From the present work, it is hypothesized that, during annealing at 800 °C, the alloying elements such as Mn, Si, and O that initially segregated along the cell walls would ‘be released’ and contribute to the nucleation of new particles and the coarsening of pre-existent oxide particles. Additionally, heat treatment at 800 °C-2 h resulted in precipitation of the σ phase, predominantly along the grain boundaries (yellow arrow in Figure 3(M3)). Multiple reports have reported the precipitation of the σ phase at 800 °C for various holding times in LPBF 316L SS. For example, Yin et al. [25] observed this phase after 30 min, Chao et al. [24] and Roirand [16] after 2 h, and Kurzynowski et al. [31] after 5 h. Thermodynamic calculation performed by Yin et al. [25] predicted that the pre-existence of Mo and the Cr segregation along the grain boundaries in the as-built condition of LPBF 316L SS facilitates σ phase formation during the annealing treatment at 600–900 °C. The local concentration of Cr and Mo, which is required to form the σ phase, may come from the dissolution of segregated cell walls at this temperature and by elemental diffusion toward grain boundaries [16]. Being hard and brittle, the σ phase can contribute to the strength of the alloy by retarding dislocation motion. On the other hand, its presence has been reported to significantly reduce the ductility of LPBF 316L SS [16,24].

In spite the presence of hard oxide inclusions and the σ phase throughout the matrix (Figure 3) in the 800 °C-2 h condition, its hardness is lower than those of the as-built and the 500 °C-2 h samples. This underpins the crucial role of the cell structure, with its elemental segregation and high dislocation densities at the cell walls and in the interior, on the superior strength of LPBF 316L SS.

## 5. Conclusions and Outlook

The present work investigated the fine-scale microstructures along the build height of LPBF 316L stainless steel (SS) and their influence on its thermal stability and mechanical properties. Along the build height, three regions were considered, namely, the top (T), middle (M), and bottom (B), and the thermal stability of the dislocation cell structure was tested after heat treatments at 500 °C and 800 °C for a dwell time of 2 h, respectively. The main conclusions are summarized as follows:

- A clear cell structure is observed in the microstructure of LPBF 316L SS. In the as-built condition, both the cell walls and cell interior have high dislocation densities at the bottom, while far fewer interior dislocations are present at the top and middle. It is suggested that this is related to the steeper thermal gradient present near the build plate during the printing of the first layers of the samples.
- Upon post-print annealing at 500 °C, no noticeable change in the cell structure is observed, which documents the high thermal stability of the dislocation structure.
- After 800 °C-2 h annealing, most of the cell structure is dissolved, although in the bottom part of the build, the cells are still visible. It is suggested that annealing for longer time or at a higher temperature would lead to the total dissolution of the cell structure.
- Spherical Mn- and Si-enriched oxide inclusions are observed in the as-built samples. After annealing at 800 °C-2 h, these oxide inclusions increase in numbers and coarsen. This may be associated with the diffusion of pre-existing alloying elements such as Mn, Si, and O out of cell walls. In addition, the non-spherical σ phase precipitated along the grain boundaries and their formations is associated with the presence of Mo and Cr at the grain boundaries.
- The microhardness decreases along the build height for all the conditions studied and is significantly harder at the bottom than at the top and middle. This agrees well with the higher dislocation density at the bottom.
- The synergetic effect of the loss of elemental segregation and the dissolution of the dislocation cell structure contribute to the considerable drop in the hardness upon heat treatment at 800 °C for 2 h.

The present work suggests that microstructural heterogeneity may be inherent to the LPBF process and that it has a considerable influence on thermal stability and mechanical properties. Strategies to mitigate microstructural heterogeneity along the build height are as follows: (i) heating the build plate to reduce the steep thermal gradient there and (ii) using support structures to minimize the effects of a ‘cold’ build plate in the bottom layers.

In the larger perspective, it is clear that microstructures in the LPBF samples and components are likely to be not only complex but also inhomogeneous, leading to anisotropy in the mechanical as well as the physical properties. This may be an advantage for certain applications, like in composites where one may obtain different strengths in different directions. Likewise, for some alloys, one may be able to tailor the phase composition to be magnetic in certain positions and not in others [32]. For other applications, however, the sample/component should be homogeneous. So far, the huge potential of microstructural engineering, used for decades in conventional manufacturing, has not been systematically explored for additive manufacturing (AM) using LPBF, but the focus is currently starting to go in that direction (see [33]). Microstructural engineering may be used to tailor the entire microstructure as well as its heterogeneity. In order for this to happen, there is a need for proper microstructural characterization on all relevant scales. By linking such experimental observations to advanced AM models, which also include microstructure simulations, the hope is that a theoretical framework can be formulated by which microstructural engineering can be performed based on physics and not just trial-and-error. Whereas such frameworks are well-established for conventional manufacturing, additional challenges in AM are that voids are hard to avoid and local stresses are always present. Incorporating these aspects into AM microstructural engineering represents a major fundamental challenge but also opens new design opportunities.

## Figures and Tables

**Table 1:** Chemical composition of 316L SS powder as provided by the supplier.

Element	Fe	Cr	Ni	Mo	Mn	Si	N	O	C
Wt.%	Balance	16.6	12.3	2.5	1.4	0.7	0.08	0.05	0.008

**Table 2:** LPBF parameters used for the present build.

Parameters	Laser Power, W	Spot Size, µm	Scanning Speed, mm/s	Hatch Spacing, µm	Layer Thickness, µm	Rotation per Layer	Volumetric Energy Density, J/mm^3^
Value	250	90	650	100	50	67°	76.9

**Table 3:** Microstructural features along the build height in the as-built, 500 °C-2 h, and 800 °C-2 h conditions.

Location Along the Build Height	Average of Mean Cell Size, nm		Average Diameter of Oxide Particles, nm		
	AB	500 °C-2 h	AB	500 °C-2 h	800 °C-2 h
Top	385 ± 14	326 ± 23	54 ± 15	51 ± 13	100 ± 49
Middle	366 ± 30	351 ± 33	50 ± 17	46 ± 15	87 ± 38
Bottom	314 ± 34	353 ± 21	51 ± 17	51 ± 14	99 ± 42

## Data Availability

The original contributions presented in this study are included in the article. Further inquiries can be directed to the corresponding authors.

## References

[1] DebRoy T., Wei H.L., Zuback J.S., Mukherjee T., Elmer J.W., Milewski J.O., Beese A.M., Wilson-Heid A.D., De A., Zhang W. (2018). Additive Manufacturing of Metallic Components–Process, Structure and Properties. Prog. Mater. Sci..

[2] Wang Y.M., Voisin T., McKeown J.T., Ye J., Calta N.P., Li Z., Zeng Z., Zhang Y., Chen W., Roehling T.T. (2018). Additively Manufactured Hierarchical Stainless Steels with High Strength and Ductility. Nat. Mater..

[3] Wang X. (2024). Cell Structure in Steels Induced by Additive Manufacturing. Mater. Sci. Technol..

[4] Funch C.V., Grumsen F.B., da Silva Fanta A.B., Christiansen T.L., Somers M.A.J. (2023). Thermal Stability of Hierarchical Microstructural Features in Additively Manufactured Stainless Steel. Heliyon.

[5] Wang X., Nadimpalli V.K., Tiedje N.S., Juul Jensen D., Yu T. (2024). Additive-Manufacturing-Induced Cell Structure in Stainless Steel 316L: 3D Morphology and Formation Mechanism. Metall. Mater. Trans. A.

[6] Pham M.-S., Dovgyy B., Hooper P.A., Gourlay C.M., Piglione A. (2020). The Role of Side-Branching in Microstructure Development in Laser Powder-Bed Fusion. Nat. Commun..

[7] Bertsch K.M., De Bellefon G.M., Kuehl B., Thoma D.J. (2020). Origin of Dislocation Structures in an Additively Manufactured Austenitic Stainless Steel 316L. Acta Mater..

[8] Kong D., Dong C., Ni X., Liang Z., Man C., Li X. (2020). Hetero-Deformation-Induced Stress in Additively Manufactured 316L Stainless Steel. Mater. Res. Lett..

[9] Kong D., Dong C., Wei S., Ni X., Zhang L., Li R., Wang L., Man C., Li X. (2021). About Metastable Cellular Structure in Additively Manufactured Austenitic Stainless Steels. Addit. Manuf..

[10] Saeidi K., Gao X., Zhong Y., Shen Z.J. (2015). Hardened Austenite Steel with Columnar Sub-Grain Structure Formed by Laser Melting. Mater. Sci. Eng. A.

[11] Saeidi K., Kvetková L., Lofaj F., Shen Z. (2015). Austenitic Stainless Steel Strengthened by the in Situ Formation of Oxide Nanoinclusions. Rsc Adv..

[12] Liu L., Ding Q., Zhong Y., Zou J., Wu J., Chiu Y.-L., Li J., Zhang Z., Yu Q., Shen Z. (2018). Dislocation Network in Additive Manufactured Steel Breaks Strength–Ductility Trade-Off. Mater. Today.

[13] Wang W.Y., Liu W., Godfrey A. (2024). Influence of Print-Chamber Oxygen Content on the Microstructure and Properties of 3D-Printed 316L. IOP Conf. Ser. Mater. Sci. Eng..

[14] Wang X., Nadimpalli V.K., Jensen D.J., Yu T. (2024). Multiscale Characterization of the Additive-Manufacturing-Induced Cell Structure in 316L Stainless Steel: A Comparative Study. IOP Conf. Ser. Mater. Sci. Eng..

[15] Voisin T., Forien J.-B., Perron A., Aubry S., Bertin N., Samanta A., Baker A., Wang Y.M. (2021). New Insights on Cellular Structures Strengthening Mechanisms and Thermal Stability of an Austenitic Stainless Steel Fabricated by Laser Powder-Bed-Fusion. Acta Mater..

[16] Roirand H., Pugliara A., Malard B., Hor A., Saintier N. (2024). Multiscale Study of Additively Manufactured 316 L Microstructure Sensitivity to Heat Treatment over a Wide Temperature Range. Mater. Charact..

[17] Fan J., Zhu Y., Wang W., Chen K., Godfrey A., Yu T., Huang X. (2024). Recovery of Dislocation Cell Structures in 316L Stainless Steel Manufactured by Selective Laser Melting. J. Mater. Res. Technol..

[18] Kjer M.B., Nadimpalli V.K., Budden C.L., Pedersen D.B. (2024). Applying Systems Engineering Principles to Develop an Open Source Laser Based Metal Powder Bed Fusion System. Rapid Prototyp. J..

[19] Kjer M.B., Pan Z., Nadimpalli V.K., Pedersen D.B. (2023). Experimental Analysis and Spatial Component Impact of the Inert Cross Flow in Open-Architecture Laser Powder Bed Fusion. J. Manuf. Mater. Process..

[20] Collins T.J. (2007). ImageJ for Microscopy. Biotechniques.

[21] Christiansen G., Bowen J.R., Lindbo J. (2002). Electrolytic Preparation of Metallic Thin Foils with Large Electron-Transparent Regions. Mater. Charact..

[22] (1999). Standard Test Method for Microindentation Hardness of Materials.

[23] (2004). Standard Test Methods for Determining Average Grain Size.

[24] Chao Q., Thomas S., Birbilis N., Cizek P., Hodgson P.D., Fabijanic D. (2021). The Effect of Post-Processing Heat Treatment on the Microstructure, Residual Stress and Mechanical Properties of Selective Laser Melted 316L Stainless Steel. Mater. Sci. Eng. A.

[25] Yin H., Song M., Deng P., Li L., Prorok B.C., Lou X. (2021). Thermal Stability and Microstructural Evolution of Additively Manufactured 316L Stainless Steel by Laser Powder Bed Fusion at 500–800 °C. Addit. Manuf..

[26] Tascioglu E., Karabulut Y., Kaynak Y. (2020). Influence of Heat Treatment Temperature on the Microstructural, Mechanical, and Wear Behavior of 316L Stainless Steel Fabricated by Laser Powder Bed Additive Manufacturing. Int. J. Adv. Manuf. Technol..

[27] Chen C., Chang S., Zhu J., Xiao Z., Zhu H., Zeng X. (2020). Residual Stress of Typical Parts in Laser Powder Bed Fusion. J. Manuf. Process..

[28] Saeidi K., Gao X., Lofaj F., Kvetková L., Shen Z.J. (2015). Transformation of Austenite to Duplex Austenite-Ferrite Assembly in Annealed Stainless Steel 316L Consolidated by Laser Melting. J. Alloys Compd..

[29] Deng P., Yin H., Song M., Li D., Zheng Y., Prorok B.C., Lou X. (2020). On the Thermal Stability of Dislocation Cellular Structures in Additively Manufactured Austenitic Stainless Steels: Roles of Heavy Element Segregation and Stacking Fault Energy. JOM.

[30] Deng P., Karadge M., Rebak R.B., Gupta V.K., Prorok B.C., Lou X. (2020). The Origin and Formation of Oxygen Inclusions in Austenitic Stainless Steels Manufactured by Laser Powder Bed Fusion. Addit. Manuf..

[31] Kurzynowski T., Gruber K., Stopyra W., Kuźnicka B., Chlebus E. (2018). Correlation between Process Parameters, Microstructure and Properties of 316 L Stainless Steel Processed by Selective Laser Melting. Mater. Sci. Eng. A.

[32] Freeman F.S.H.B., Lincoln A., Sharp J., Lambourne A., Todd I. (2019). Exploiting Thermal Strain to Achieve an In-Situ Magnetically Graded Material. Mater. Des..

[33] Alphonso W.E., Defer M., Godfrey A., Juul Jensen D., Mohanty S., Nadimpalli V.K., Pan Z., Pantleon W., Yu T., Zhang Y. (2024). 44th Risø International Symposium on Materials Science (Riso 2024).

